# Reference Values of Native T1 at 3T Cardiac Magnetic Resonance—Standardization Considerations between Different Vendors

**DOI:** 10.3390/diagnostics11122334

**Published:** 2021-12-11

**Authors:** Liliana Tribuna, Pedro Belo Oliveira, Alba Iruela, João Marques, Paulo Santos, Tiago Teixeira

**Affiliations:** 1 Department of Radiology, Hospital da Luz Aveiro, 3800-009 Aveiro, Portugal; pedro.nunobelooliveira@hospitaldaluz.pt (P.B.O.); joao.oliveira.marques@hospitaldaluz.pt (J.M.); pauloalexandrealmeida.santos@hospitaldaluz.pt (P.S.); tiagoteixeiranoc@gmail.com (T.T.); 2 Department of Radiology, Centro Hospitalar Universitário de Coimbra, 3004-561 Coimbra, Portugal; 3 Clinical Scientist in MR, Canon Medical Systems Spain and Portugal, 08940 Cornellà de Llobregat, Spain; alba.iruela@eu.medical.canon; 4 Department of Cardiology, Centro Hospitalar de Entre o Douro e Vouga, 4520-211 Santa Maria da Feira, Portugal

**Keywords:** cardiac MR, native T1 mapping, MOLLI

## Abstract

This study aimed at establishing native T1 reference values for a Canon Vantage Galan 3T system and comparing them with previously published values from different vendors. A total of 20 healthy volunteers (55% Women; 33.9 ± 11.1 years) underwent left ventricular T1 mapping at 3T MR. A MOLLI 5(3)3 sequence was used, acquiring three short-axis slices. Native T1 values are shown as means (±standard deviation) and Student’s independent samples *t*-test was used to test gender differences in T1 values. Pearson’s correlation coefficient analysis was used to compare two processes of T1 analysis. The results show a global native T1 mean value of 1124.9 ± 55.2 ms (exponential analysis), that of women being statistically higher than men (1163 ± 30.5 vs. 1077.9 ± 39.5 ms, respectively; *p* < 0.001). There were no specific tendencies for T1 times in different ventricular slices. We found a strong correlation (0.977, *p* < 0.001) with T1 times derived from parametric maps (1136.4 ± 60.2 ms). Native T1 reference values for a Canon 3T scanner were provided, and they are on par with those already reported from other vendors for a similar sequence. We also found a correlation between native T1 and gender, with higher values for women.

## 1. Introduction

Cardiac magnetic resonance (CMR) is considered the gold standard for ventricular volume and function determination, but the advantages of the technique span from anatomic to tissue characterization, perfusion and viability [1,2]. Therefore, CMR has been progressively used for clinical cardiology, being helpful in the diagnosis of ischemic and congenital heart diseases, cardiomyopathies and myocarditis [3].

As a state-of-the-art method, CMR is continuously evolving, and its tissue quantification techniques, such as T1 mapping, are among those most unanimously lately adopted by the scientific community [4]. This technique involves multiple acquisitions of T1 weighted images, whose signal intensities are fitted to an MR sequence model that describes the T1 relaxation curve [5]. T1 mapping specifically refers to the pixelwise quantification of the myocardial longitudinal T1 relaxation values, and it can be accomplished by using different sequences [3,6]. The ones with greater clinical experience are based on a Look-Locker inversion recovery [5,7], the most widely applied protocols being the modified Look-Locker imaging (MOLLI) [8] and shortened MOLLI (ShMOLLI) [9]. The original MOLLI implementation requested 17 heartbeats for each breath hold in three sets of acquisitions separated by two breaks (described in brackets), denoted as 3(3)3(3)5 [8]. Several variations have been proposed, such as 5(3)3 [10], which is more insensitive to heart rate variations, and 4(1)3(1)2 [11], which is applicable especially after gadolinium contrast when extracellular volume is also being determined [12]. The saturation-recovery single-shot sequences (SASHA) [13] are an alternative to inversion-recovery acquisitions that use a saturation recovery instead, conveying in accuracy what they may lack in image quality or precision [14].

T1 mapping contributes to the non-invasive characterization of the myocardial health state, which is helpful to identify abnormalities in the context of both acute and chronic events, such as edema and fibrosis, as well as detection of fat, iron and amyloid accumulation [3,6,15]. Typically, native T1 times are longer with interstitial expansion caused by edema, infarction, amyloid infiltration and fibrosis. On the other hand, fat and iron accumulation results in shortened T1 times [6,16]. The specific abnormal values, especially those of some diseases (e.g., amyloidosis), are easily differentiated from healthy ones, but the cut-offs are difficult to define. Naturally, that process begins with the determination of the normal ranges. The difficulty is that, although native T1 values are reproductible, they vary due to several reasons, namely magnetic strength (3T resulting in longer native T1s than 1.5T), vendor platforms and the acquisition sequence [16]. Therefore, before clinical use, a local data set should be collected, with the same features of the planed application, to define the normal ranges of native T1 values, and local results should preferably be benchmarked against published reports [17].

The aim of this research was to determine the native T1 values for a population of healthy individuals with our 3T MR scanner, comparing them with the published values of different vendors, more widely reported so far.

## 2. Materials and Methods

The research was performed at Hospital da Luz Aveiro (Aveiro, PT), using a 3T clinical MR scanner (Vantage Galan 3T/SGO, Canon Medical Systems Corporation, Tochigi, Japan). Healthy volunteers were screened from local publishing. After signing the internal consent form, approved by the institution’s ethics committee, non-contrast cardiac MR was performed between July 2019 and September 2021 (the prolonged recruitment was impacted by the COVID-19 pandemic). The confidentiality and anonymity of all volunteers was guaranteed, and any abnormal findings on the images, cardiac or extra cardiac, were conveyed to the participants.

### 2.1. Study Population

CMR was performed on 22 volunteers without main cardiovascular disease risk factors (smoking, high blood cholesterol, arterial hypertension, *diabetes* mellitus and family history of premature cardiovascular disease, that is ≤55 years for men and ≤65 years for women).

### 2.2. Cardiac MR Protocol

All images were acquired on a Canon Vantage Galan 3T scanner with combined 16-channel anterior (body coil) and 40-channel posterior (spine coil) coils. All participants were scanned using the same CMR protocol, as shown in Table 1. The parameters of the sequences can also be seen in that table.

### 2.3. T1 Mapping Analysis

The parametric T1 map’s quality can be affected by many artifacts, such as motion (respiratory and cardiac), miss-triggering and magnetic susceptibility [18]. In accordance with the recommendations of the Society for Cardiovascular Magnetic Resonance (SCMR), a visual assessment of the images was performed, ensuring their diagnostic quality [18,19].

**Figure 1:**
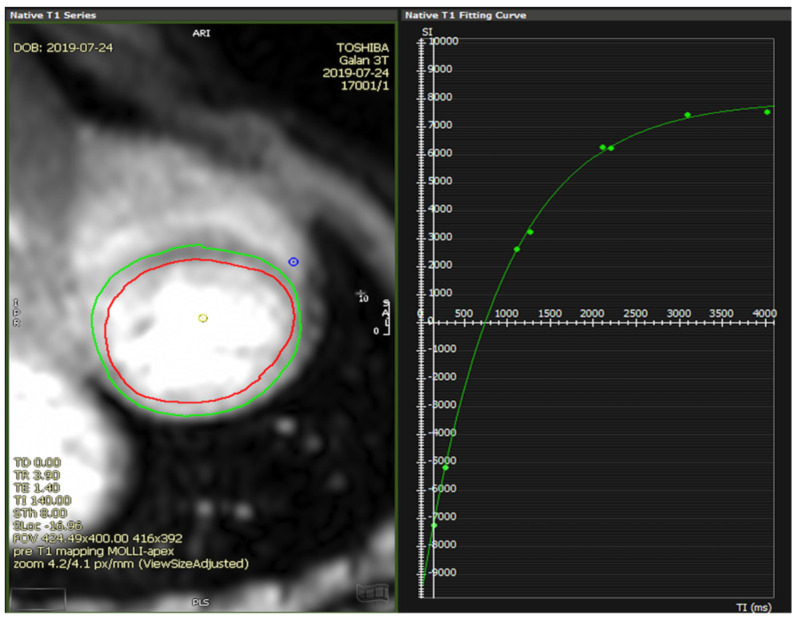
Exponential determination of T1 relaxation times.

**Figure 2:**
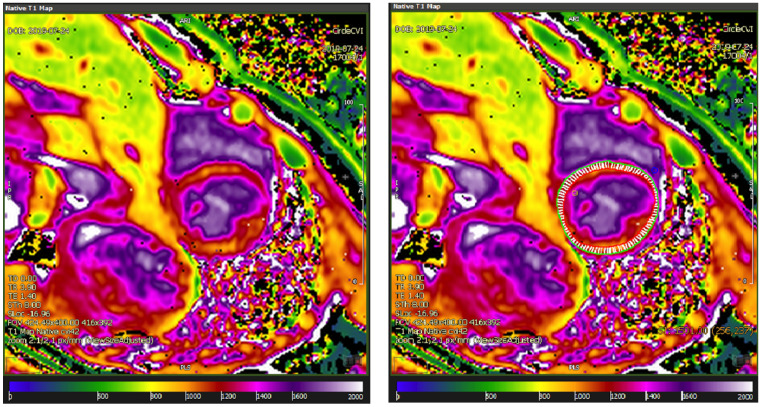
Parametric T1 maps from midventricular slice, without (**left**) and with (**right**) T1 determination.

The images’ post processing was performed using Circle Cardiovascular Imaging (CVI^42^) software, client version 5.12.4, Canada, and they were analyzed in two ways:

- By drawing a ROI in the area of interest. We preferred using endocardial and epicardial contours for the entire slice being analyzed; afterwards, the ROIs were forwarded to all the 8 images, with eventual adjustments in their position depending on respiratory variation between images, and the software returned the T1 values (ms), according to the exponential curve of the respective type of T1 mapping sequence (Figure 1) [7,18,19,20].
- By acquiring a co-registered image set, where the T1 value of each pixel was encoded to display a color. Thus, a parametric T1 map was created by the analysis software (in this case, CVI^42−^ derived), allowing further qualitative assessment but also containing pixel-wise information of the T1 values (Figure 2, right panel) [6,7,12,14].

Quantitative analysis was performed by a reader with 10 years of CMR experience.

### 2.4. Statistical Analysis

From previous reported native T1 values at 3T from other vendors, we estimated that a sample size of 16 subjects would have the power to determine the mean T1 of our population, without finite population correction: *n* = Inf, precision error = 25 and standard deviation S = 50 [21].

The collected data was analyzed by using SPSS statistics, IBM, software version 25.0. Continuous variables are presented as means +/− standard deviation (SD) and were compared using Student´s independent samples t-test. Categorical variables were expressed as a percentage. Pearson’s correlation coefficient analysis was used to compare native T1 values estimated from exponential and map analysis. Univariate predictors of native myocardial T1 times were assessed with a multiple linear regression model that included age, gender and left ventricular ejection fraction. A two-sided *p*-value inferior to 0.05 indicated a statistically significant result.

## 3. Results

A total of 22 volunteers completed the study protocol, although after image analysis, two volunteers were excluded, one due to a mild pericardial effusion and the other due to severely impaired T1 mapping evaluation due to artifacts. Thus, our study population consisted of 20 volunteers, with a mean age of 33.9 ± 11.1 years, and 55% of participants were women (*n* = 11). Demographic and CMR gender characteristics are compared in Table 2.

Women had smaller ventricles on systole (23.1 ± 3.7 vs. 30.6 *±* 10.6, *p* *<* 0.05), with lesser myocardial mass (44.0 *±* 10.1 g vs. 58.4 *±* 7.1 g, *p <* 0.05). For the remaining features there were no gender differences on the overall population. Global myocardial T2 in relation to striated muscle was 1.2 *±* 0.3 ms, translating to the absence of myocardial edema.

In Table 3, the native myocardial T1 values for the overall population are shown, as well as gender differences.

**Figure 3:**
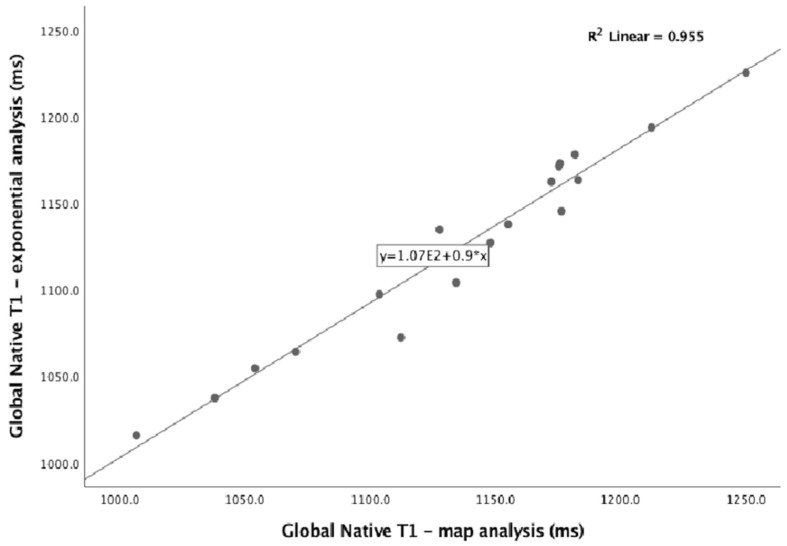
Correlation between T1 values acquired by map analysis and values acquired by exponential analysis.

The overall population had a mean of global native T1 of 1124.9 *±* 55.2 ms. There were statistically significant differences between women and men for all the displayed myocardial slices, women having higher mean global native T1 times (1163.4 *±* 30.5 ms vs. 1077.9 *±* 39.5 ms, *p <* 0.001), with no specific tendencies on the different ventricular slices. T1 times derived from map analysis (1136.4 *±* 60.2 ms) showed good correlation with the those from exponential analysis (0.977, *p* < 0.001)), with similar gender differences observed (Figure 3). Gender remained the only determinant of T1 times on a multiple linear regression model that also included age and LVEF.

## 4. Discussion

We analyzed the native T1 values of a population of healthy individuals with the 3T clinical MR scanner at the Hospital da Luz Aveiro, aiming to establish the native T1 reference values at our institution. To our knowledge, this is the first publication on native T1 reference values from a Canon 3T scanner.

As reported in previous publications, different T1 mapping sequences and field strengths are associated with some variation in normal values [22]. There are multiple factors that can contribute to that variability, such as the vendor, the scanner field strength and the T1 mapping sequence, as well as covariants related to the patient, such as age, gender and hematocrit value [23]. A demographic data evaluation in this study showed that women had smaller LVMM than men (44 *±* 10 vs. 58 *±* 7 g/m^2^, respectively). This difference was in agreement with the results found by both Roy et al. [24] (51 ± 10 g/m^2^ vs. 64 ± 9 g/m^2^, respectively) and Dong et al. [25] (42 ± 7 g/m^2^ vs. 50 ± 10 g/m^2^, respectively). Reference ranges for cardiac structure and function using CMR were published by Petersen et al. [2]. Values of LVMM of 29–55 g/m^2^ for women and 35–70 g/m^2^ for men were reported as normal, and our results were in agreement with this reference.

Over the past few years, many studies have been published to establish native T1 value references for both 1.5T and 3T MR. We had previously reported healthy volunteers’ native T1 values, as controls of larger population data, by using MOLLI sequences at Siemens 3T with a 3(3)3(3)5 sequence [26]; A total of 10 volunteers had a mean of 1219 ± 61 ms with a 5(3)3 sequence [7] and 9 volunteers had a mean of 1208 ± 18 ms. Dong et al. [25] had similar values in 69 volunteers (1202 ± 45 ms), also using a Siemens MOLLI 5(3)3 but using a higher flip angle (50°). Granitz et al. [22] and Roy et al. [24], carried out studies with Philips scanners with a 5(3)3 (1184 ± 38 ms, 58 volunteers) and a 3(3)3(3)5 (1122 ± 57 ms, 75 subjects) MOLLI, both with a 35° flip angle. However, Roy’s et al.’s [24] population included several individuals with cardiovascular risk factors. As a clinical biomarker, native T1 myocardial values can better define healthy individuals if a range of normal values is established. Due to the referred variation in T1 values, these ranges can be defined through tolerance intervals. Tolerance intervals predict that, according to a certain confidence level, we can be sure that a percentage of the population will be contained in the interval (using average k*StDev, where k is a tabled value based on the sample size and confidence level) [21]. If we look at the studies that used the exact same sequence as this study and calculate their tolerance intervals, we find that there is overlaying of the values, as shown in Table 4, and therefore they are not significantly different.

The characteristics of the T1 mapping sequence such as TE, TR and FA were reported as capable of modifying T1 values [27]. Popescu et al. [28] recently performed a systematic review of literature and meta-analysis of published native T1 values, derived from MOLLI sequences. At 3T, only the values of the two aforementioned vendors were available to analyze. Interestingly, they performed clustering of the values, along with patterns of such sequence physic characteristics. The most prevalent cluster (TE 1.07, TR 2.14, FA 35°, 330 subjects) showed mean values of 1160 ± 21 ms. The values from this study were in the range of these reported values. However, the cluster with a higher FA at 3T resulted in a lower T1. This does not occur with the sequence used in this study, as our 13˚ FA generates values in the lower range of these reported T1s, especially for men. The choice for such a FA intends for less off-resonance artifacts and a lower heart rate variability, along with a specific absorption rate (SAR).

The correlation between native T1 values and gender has been widely studied, and it is known that, usually, native T1 values are higher in women than men [29]. The results from this study are consistent with this phenomenon, with means of 1164 ± 31 ms for women and 1078 ± 40 ms for men, in agreement with Roy et al. [24], Dong et al. [25] and Rosmini et al. [30] studies, even though the latter estimated values of 1.5 T. One of the hypotheses is that a lower hematocrit in child-bearing-aged women conveys higher T1s [31]. However, Dabir et al. [32] showed no correlation between gender and native T1 values, using a 3(3)3(3)5 MOLLI in 102 subjects at 3T Philips. Both Roy et al. [24] and Dong et al. [25] also studied the correlation between native T1 values and age. Roy et al. [24] reported that T1 values increase in men with age, but the same did not occur in women, while Dong et al. [25] did not find significant differences in myocardial native T1 values between older and younger groups. Despite having a smaller sample size, did not find a correlation with age either.

For this study three slices were acquired at the base, midventricular level and apex of the left ventricle to evaluate possible variations of T1 values according to slice location [7,32]. Reiter et al.’s [29] systematic review suggested that the apical native T1 values tend to be slightly higher compared to the basal and midventricular slices. This trend was not seen in our research, making such inferences impossible. Furthermore, Von Knobelsdorff-Brenkenhoff et al. [33] did not observe this trend either, reporting higher values for the midventricular zone. This discrepancy among the results may result from the sensitivity of T1 mapping sequences to many factors but also from the contouring method, with the possibility of wrongly including partial volume regions in some of the apical thinner ROIs.

The results from this study show good correlation between the T1 values derived from the exponential analysis and the map analysis (derived from CVI^42^ software). This is extremely important from a clinical perspective, because native T1 values are normally complemented by the acquisition of late post-contrast ones to compute extracellular volume (ECV) fraction [31] in a regular exam. Although ECV can also be manually calculated, if the hematocrit and the intensity of the blood pool are known, computing it by using both native and post-contrast maps enables the construction of a respective ECV map that renders qualitative as well as quantitative analysis, englobing the advantages of both T1 estimation and late gadolinium enhancement evaluation. ECV is also less sensitive to different field strengths [31]. However, as we have advanced in the past [7], the exponential estimation of T1 allows for the correction of motion and cardiac phase artifacts from image to image, which is impossible when using the map analysis. Therefore, extra care is necessary to reduce any of these artifacts and consequently to be able to use T1 and ECV maps. Tao et al. [4] demonstrated that the process of ROI identification can in turn be automatized, with fast performing, high accuracy and results highly correlated to those obtained from manual annotation.

Despite all these attempts to standardize T1 values across sequences and vendors, due to intra and interpatient variability, the SCMR recommended that institutions establish optimized protocols for acquisition of T1 mapping images, reducing the unwanted variability of T1 values as a result of the variability of image acquisition [17]. There is still a long way to go in the standardization of native T1 values. To achieve this, there must be a shared effort among the vendors and the scientific community to create protocols of acquisition that reduce variability and increase the consistency of native T1 values.

Our study had some limitations. The study population was small, although it surpassed our estimation size. However, due to its high precision, cardiac MR offers the advantage to find big differences even in small groups. Furthermore, this sample’s gender distribution was balanced, and age distribution also embraced an interesting part of a healthy age range (19 to 66 years). Intra- or inter-reader reproducibility was not performed. Although that type of analysis could have strengthen the results, the native T1 values reported here only constitute general indication, as we recommend that the normal ranges are performed in every institution and scanner. Therefore, we used the analysis of the clinical reader in our institution. In future work it will be important to report values from different groups with cardiac disease from the vendor.

## 5. Conclusions

This study provides first reference values of native T1 for healthy individuals for MOLLI at a Canon Vantage Galan 3T, demonstrating that these results were on par with others previously published from different vendors. Nevertheless, native T1 values are influenced by several factors, and definition of local normal range values is encouraged, preferably with robust-sized samples. These values demonstrated gender dependency, with higher values for women, and it is necessary to take this into account in interpretation and clinical decision. Future standardization of T1 mapping protocols is warranted, as it could reduce some of the variability of values between scanners or even between vendors. Professional societies should work in collaboration with all the vendors to try further regulate those protocols.

## Figures and Tables

**Table 1:** Typical sequence parameters of the CMR protocol performed on all participants of the study.

	Base Sequence	TR (ms)	TE (ms)	FA (°)	FOV (mm)	Matrix	Slice Thickness (mm)	Gap (mm)	Number of Slices	BW (Hz)
T2 AX	FASE	9100	80	90	450 × 340	224 × 256	8	2	16–18 (Aortic arch to liver)	651
Cine 2ch	SSFP	2	1.5	55	400–450 × 400–420	224 × 192	8	2	1	1302
Cine 4ch									1	
Cine SA									9–11 (depending on heart size)	
T2 FS AX	FSE	2036	60	90	400–450 × 300	160 × 256	8	6–8	3 (base, midventricular and apex)	651
T1 mapping	MOLLI 5(3)3	3.9	1.4	13	400 × 450	128 × 208	8	2	3 (base, midventricular and apex)	408

TR: repetition time; TE: echo time; FA: flip angle; FOV: field of view; BW: bandwidth; FASE: fast advanced spin echo sequence; SSFP: steady-state free precession sequence; FSE; fast spin echo sequence.

**Table 2:** Demographic and CMR gender characteristics of study population.

	Overall Population (*n* = 20)	Women (*n* = 11, 55%)	Men (*n* = 9, 45%)	*p* Value *
Age (years)	33.9 ± 11.2	32.1 ± 10.0	36.1 ± 12.7	0.440
LVEDV (mL/m^2^)	75.6 ± 17.7	66.7 ± 8.4	75.6 ± 17.7	0.160
LVESV (mL/m^2^)	30.6 ± 10.6	23.1 ± 3.7	30.6 ± 10.6	0.040
LVMM (g/m^2^)	58.4 ± 7.1	44.0 ± 10.1	58.4 ± 7.1	0.002
LVEF (%)	60.6 ± 6.1	65.1 ± 5.3	60.6 ± 6.1	0.090
T2	1.3 ± 0.3	1.2 ± 0.2	1.3 ± 0.3	0.550

LVEDV: Left Ventricular End Diastolic Volume Index; LVESV: Left Ventricular End Systolic Volume Index; LVMM: Left ventricular myocardial mass; LVEF: Left ventricular ejection fraction; * *p* value for women and men comparison.

**Table 3:** Native T1 values estimates.

T1 Estimates	Overall Population	Women	Men	*p* Value *
Global (ms)	1124.9 *±* 55.2	1163.4 *±* 30.5	1077.9 *±* 39.5	<0.001
Base (ms)	1133.0 *±* 49.1	1161.9 *±* 35.8	1097.6 *±* 39.4	0.001
Midventricular (ms)	1119.5 *±* 62.0	1160.0 *±* 35.2	1069.9 *±* 50.4	<0.001
Apex (ms)	1125.9 *±* 64.5	1168.3 *±* 37.0	1067.8 *±* 45.3	<0.001

* *p* value for Women and Men.

**Table 4:** Tolerance intervals in the studies using a MOLLI 5(3)3 sequence.

Study	*n*	Native T1	Tolerance Interval (80% Confidence to Include 95% of the Population)
Teixeira et al. [7] Siemens	9	1208 ± 18 ms	1170.8–1245.0
Dong et al. [25] Siemens	69	1202 ± 45 ms	1150.4–1253.6
Granitz et al. [22] Philips	58	1184 ± 38 ms	1139.4–1228.6
Tribuna et al. Canon	20	1125 ± 55 ms	1042.4–1207.4

*n* = sample size.

## Data Availability

Not applicable.
